# Vertical La_0.7_Ca_0.3_MnO_3_ nanorods tailored by high magnetic field assisted pulsed laser deposition

**DOI:** 10.1038/srep19483

**Published:** 2016-01-18

**Authors:** Kejun Zhang, Jianming Dai, Xuebin Zhu, Xiaoguang Zhu, Xuzhong Zuo, Peng Zhang, Ling Hu, Wenjian Lu, Wenhai Song, Zhigao Sheng, Wenbin Wu, Yuping Sun, Youwei Du

**Affiliations:** 1 Key Laboratory of Materials Physics, Institute of Solid State Physics, Hefei Institutes of Physical Science, Chinese Academy of Sciences, Hefei, 230031, China; 2 High Magnetic Field Laboratory, Chinese Academy of Science, Hefei, 230031, China; 3 University of Science and Technology of China, Hefei, 230026, China; 4 Nanjing National Laboratory of Microstructures and Department of Physics, Nanjing University, Nanjing 210093, P.R. China

## Abstract

La_0.7_Ca_0.3_MnO_3_ (LCMO) thin films on (LaAlO_3_)_0.3_(Sr_2_AlTaO_6_)_0.7_ (001) [LSAT (001)] single crystal substrates have been prepared by high magnetic field assisted pulsed laser deposition (HMF-PLD) developed by ourselves. Uniformly sized and vertically aligned nanorod structures can be obtained under an applied high magnetic field above 5 T, and the dimension size of the nanorods can be manipulated by varying the applied magnetic field. It is found that the magnetic anisotropy is strongly correlated to the dimension size of the nanorods. A significantly enhanced low-field magnetoresistance (LFMR) of −36% under 0.5 T at 100 K can be obtained due to the enhanced carrier scattering at the vertical grain boundaries between the nanorods for the LCMO films. The growth mechanism of the nanorods has been also discussed, which can be attributed to the variation of deposition rate, adatom surface diffusion, and nucleation induced by the application of a high magnetic field in the film processing. The successful achievements of such vertical nanorod structures will provide an instructive route to investigate the physical nature of these nanostructures and achieve nanodevice manipulation.

Perovskite manganites, such as La_0.7_Ca_0.3_MnO_3_ (LCMO), have aroused great interest in both bulk and thin film due to their complex properties and colossal magnetoresistance (CMR) effect, which have been considered as good candidates for spintronics such as magnetic sensors, computer memory, and data storage^1^,^2^,^3^ etc. However, the practical application of the intrinsic CMR effect has been limited due to the required high magnetic field of several Tesla. In the past few years, the low-field magnetoresistance (LFMR) (*H* ≤ 1 T) effect, obtained by structuring grain boundaries (GBs), nanosized inclusions, interface phase, and artificial grain boundaries^4^, has been paid more attention. Such enhanced LFMR has been explained by the spin-polarized tunneling between adjacent grains or spin-dependent scattering at the grain boundaries^5^,^6^,^7^,^8^,^9^,^10^, and interfacial and grain boundary effects have been shown to play important roles for the enhanced LFMR. Vertical aligned nanostructures, such as nanorods and nanowires, have powerful advantages over the conventional planar film structures. The vertically aligned nanostructures have a vertical interfacial area much larger than the substrate area, and will produce additional grain boundaries, thus improving the LFMR^11^,^12^. Furthermore, magnetic anisotropy is highly dependent on the vertically aligned nanostructures due to the enhanced vertical interfacial area and vertical shape anisotropy^12^,^13^,^14^,^15^. Many groups have attempted to improve the LFMR effect and magnetic properties of manganite films by introducing vertical aligned nanostructures, which are usually achieved in manganite-based composites by incorporating a secondary phase such as ZnO^12^, NiO^16^, MgO^17^,^18^, and V_2_O_3_^19^ etc. A self-assemble columnar growth can be induced by the addition of the second phases due to a large lattice mismatch between the two phases, and between each phase and the substrate^11^,^16^,^17^. For example, Moshnyaga *et al.*^17^ showed vertical nanocolumnar structures in epitaxial LCMO: MgO nanocomposite films, and demonstrated the importance of controlling the vertical interfaces. The LFMR values of -17.5% at 30 K and 1 T in La_0.7_Sr_0.3_MnO_3_ (LSMO): ZnO nanocolumnar structure^12^, and –41% at 10 K and 1 T in LSMO: NiO nanocolumnar structure have been reported^16^. It is noted that the size of the vertical nanostructures plays a critical role in tuning the properties of manganite thin films due to the vertical interfacial and grain boundary effects. However, for the strain-driven self-assembly with well-ordered vertical grain boundaries, there are only a few routes to control and manipulate the size of vertical nanostructures, such as phase ratio, growth rate, and so on, which require sophisticated technology and stringent fabrication conditions and have been recognized as a hard-attainable issue.

The application of high magnetic fields as well as strain has a unique effect on the preparation process and physical properties of novel and functional materials acting as an external varying parameter^20^,^21^,^22^,^23^,^24^,^25^. For example, the superconducting properties of YBa_2_Cu_3_O_7_ (YBCO) bulks as well as YBCO thin films can be significantly enhanced due to the modified texture, grain size and grain orientation with the application of a high magnetic field in processing^20^,^23^. Therefore, if a high magnetic field is applied during a film fabrication, the growth kinetics, nucleation, growth mode and nanostructures would be modified, and amazing effects are expected. Recently, a high magnetic field assisted pulsed laser deposition (HMF-PLD) system have been developed by ourselves^26^, which could be used in *in-situ* growth of thin films under a high magnetic field in pulsed laser deposition processing. In this work, we use the HMF-PLD to grow LCMO thin films on (LaAlO_3_)_0.3_(Sr_2_AlTaO_6_)_0.7_ (001) [LSAT (001)] substrates. Our results show that uniformly sized, vertically aligned and one-dimensional nanorod structures can be obtained under a high magnetic field above 5 T. More interestingly, the dimension size of the nanorod structures can be tuned by the strength of the magnetic field. The associated magnetic anisotropy and LFMR are found to be highly dependent on the nanorod structures. The successful achievements of such one-dimensional nanorods will provide an opportunity to investigate the physical properties of these nanostructures and realize the nanodevice manipulation.

Figure 1 shows the schematic illustration of the HMF-PLD system. The HMF-PLD system is established by the integration of a superconducting magnet with a PLD system. A homogenous central magnetic field up to 10 T can be produced by a closed-cycled cryogen superconducting magnet with a bore diameter of 200 mm. A special PLD cylindrical vacuum chamber is horizontally located in the bore configuration of the magnet. An excimer laser with KrF gas mixture operating at the wavelength of 248 nm is employed as the deposition source.

The LCMO films were deposited on (001) oriented LSAT single crystal substrates under various high magnetic fields. The selection of LSAT substrate is attributed to the small lattice mismatch (–0.2%) between the bulk and the substrate, and vertically aligned nanorod structures are almost impossible to be obtained in PLD processing without magnetic field due to the small derived strain^27^, which is also confirmed in our experiments. The successful achievement of nanorod structures in such small lattice mismatch will provide a unique route to tune the nanostructures in the thin films on substrates with small lattice mismatch, which is impracticable in PLD processing without a magnetic field. For the LCMO films grown under different magnetic fields, it is observed that when the applied magnetic field is higher than a critical value, the vertically aligned nanorod structures will occur, and the critical magnetic field is in the range of 4–5 T. Above 5 T, vertically aligned nanorod structures are formed and the dimension size of the nanorods is gradually reduced with further increasing magnetic field. Here, a typical set of LCMO thin films are presented to give a clear image about nanostructure tailoring by HMF-PLD. The LCMO films deposited under 0, 4, 5 and 8 T are denoted as LCMO-0 T, LCMO-4 T, LCMO-5 T and LCMO-8 T, respectively. The applied magnetic fields in deposition process are denoted as *H*_*D*_, and the measured magnetic fields in magnetic property measurement are denoted as *H*_*M*_.

## Results and Discussion

**Figure 2:**
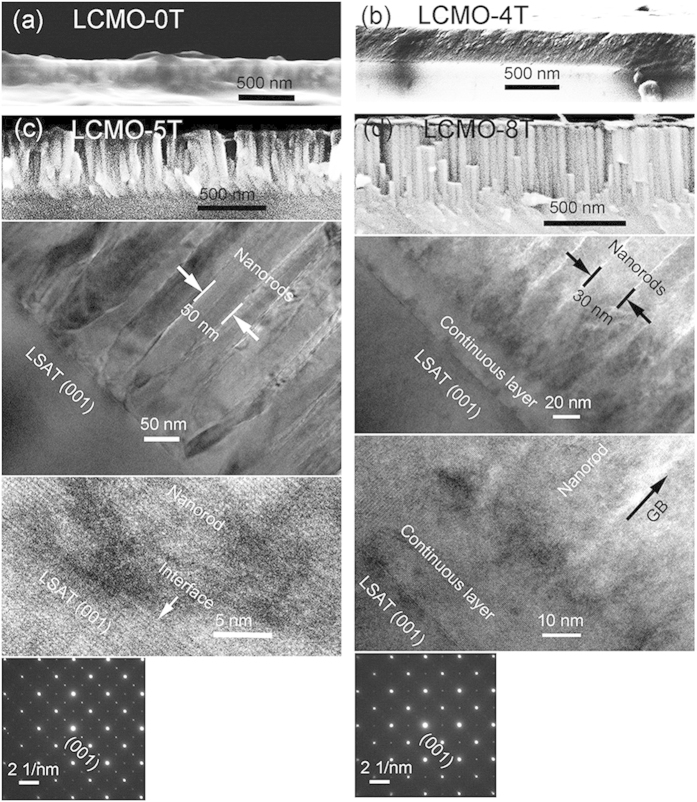
(**a**) Cross-sectional FESEM image of the LCMO-0T film; **(b)** the cross-sectional FESEM image of the LCMO-4T film; the cross-sectional FESEM, TEM, HRTEM and SAED images of **(c)** the LCMO-5T and **(d)** LCMO-8T films, respectively.

The derived LCMO thin films were characterized by field emission scanning electron microscopy (FESEM) and transmission electron microscopy (TEM). The cross-sectional FESEM and TEM images of the LCMO films are shown in Fig. 2. The LCMO-0T film shows a continuous planar film structure with a film thickness of about 220 nm (Fig. 2a), which can be considered to follow a Frank-van der Merwe layer-by-layer growth mode^28^ due to a negligible lattice mismatch. The LCMO-4T film shows an obvious increase in film thickness and the film thickness is about 320 nm (Fig. 2b). The LCMO-4T film also shows a continuous planar film structure, however, its cross-sectional morphology is different from that of the LCMO-0T film, indicating a transitional structure. The LCMO-5T film shows vertically aligned nanorod structures with an average dimension size of 50 nm. The nanorods almost initiate at the film-substrate interface extending to the top film surface, and the whole film thickness is about 460 nm, as confirmed from the FESEM, TEM and high resolution TEM (HRTEM) images (Fig. 2c). The selected-area electron diffraction (SAED) image of the LCMO-5T film from the interface region indicates that the nanorods are epitaxially grown. It is noted that there is no an obvious continuous layer for the LCMO-5T film and the highly disordered grain boundaries between the nanorods are also observed and exhibit a thickness of 5–8 nm. The LCMO-5T film can be considered to follow a Stranski-Krastanov layer-plus-island growth mode^28^. Differently, the LCMO-8T film shows a two-layer structure: a continuous thin planar epitaxial layer about 30 nm thick adjacent to the substrate, and then a nanorod layer with a length of about 480 nm, which is uniformly sized and vertically aligned extending from the bottom layer to the top surface (Fig. 2d). Each nanorod has an average dimension size of 30 nm with disordered grain boundaries between the nanorods as denoted by the arrow, which have a thickness of 6-9 nm. The SAED image of the LCMO-8 T film from an area covering both the continuous layer and nanorod layer indicates that the nanorods are also epitaxially grown on the continuous layer. These results show that the LCMO-8T film also follows layer-plus-island growth mode, with a thicker film thickness, a thinner dimension size and an additional continuous layer compared to those of the LCMO-5T film.

**Figure 3:**
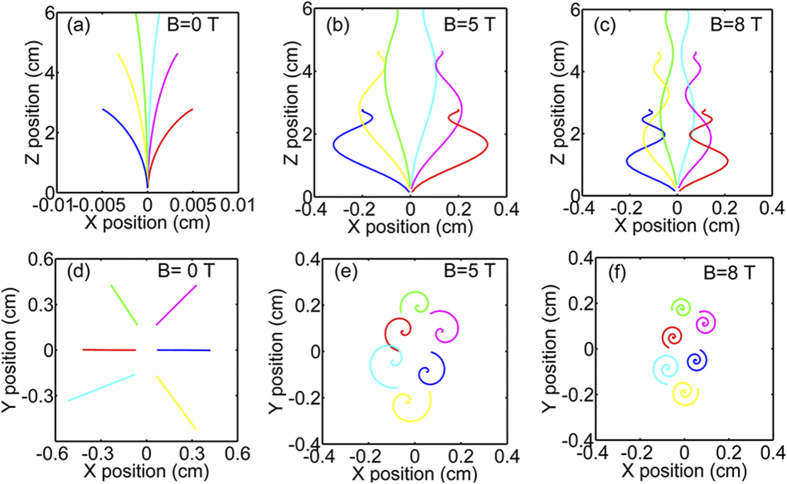
Simulations on the movement of evaporated particles in a plume under different magnetic fields: **(a)** *B* = 0 T, **(b)** *B* = 5 T and **(c)** *B* = 8 T. And simulations on adatom surface diffusion on the substrate under different magnetic fields: **(d)** *B* = 0 T, **(e)** *B* = 5 T and **(f)** *B* = 8 T. Only Lorentz force and inelastic scattering are included for the calculation.

As for the forming and tailoring of vertical nanorod structures of the derived LCMO thin films, the applied magnetic field should be the unique reason since the processing is same for all thin films except for the magnetic field. An applied magnetic field will produce a Lorentz force as well as a magnetization force. The introduction of these two forces will play crucial roles in determination of the nanostructures due to the variation of plume dynamics, adatom surface diffusion and nucleation as well as growth mode. When an applied magnetic field is normal to the substrate, the evaporated particles in a plume show spiral movements (Fig. 3a–c) due to the Lorentz force, which will lead to an increase of the deposition rate as confirmed in Fig. 2 in our experiments. On the other hand, the adatom surface diffusion on the substrate will be also constrained by the Lorentz force, which is proportional to the velocity of particles and the strength of high magnetic field (Fig. 3d–f). The constrained effect could appear strongly. For example, the Lorentz cyclotron radius of Mn^3 + ^on the substrate surface is about 0.22 mm for the particle kinetic energy *E* = 10 eV and *B* = 5 T. The confinement of surface diffusion will also accelerate the thin film growth along the out-of-plane direction of the substrate. Crystal growth, viewed on an atomic scale, consists of the creation, the migration and the annihilation of defects such as two-dimensional islands, misfit dislocations and so on. Any process modifying the distribution and dynamics of these defects will change the growth mode to a greater or lesser extent^29^. Any increase in roughness is due to the kinetic limitation^30^. As such, the confinement of surface diffusion and the increase of deposition rate can change the growth mode of thin films from a layer-by-layer growth to a layer-plus-island growth, eventually resulting in a formation of nanorod structures^31^,^32^. The increase of a deposition rate will lead to a higher nucleation site density^30^,^33^. On the other hand, magnetic anisotropy is an inherent characteristic in LCMO materials^34^. Although the LCMO materials show a paramagnetic behavior under the processing temperature, a magnetization force has to be considered under a high magnetic field due to the presence of paramagnetic anisotropy. Following classic nucleation theory, *V*_*C*_ = 2*K*_*B*_*T*/(Δ*χH*_*D*_^2^)^20^,^35^, where *V*_*C*_ is the critical volume of a nucleus, *K*_*B*_ is the Boltzmann constant, ∆*χ* is the anisotropic paramagnetic susceptibility and *H*_*D*_ is the applied magnetic field. It is clearly seen that the nucleus volume *V*_*C*_ is inversely proportional to *H*_*D*_^2^, which can also lead to a higher nucleation site density due to the reduction of critical volume^23^. A higher nucleation site density is normally associated with the growth of films composed of many smaller crystallites^30^, which results in the decrease in dimension size of nanorods with increasing magnetic field. Another consequence of a high density of nucleation is that the initial film growth is beneficial for a layer-by-layer growth^30^. Jenniches *et al.* investigated the growth of Fe on Cu using PLD and discovered that initial growth was two-dimensional due to a very high deposition rate^36^. As such, the occurrence of the continuous layer of the LCMO-8T film might be attributed to a higher nucleation site density with increasing applied magnetic field. As aforementioned, it is suggested that the combined effects of Lorentz force and magnetization force are responsible for the tuned nanorod structures in HMF-PLD processing.

**Figure 4:**
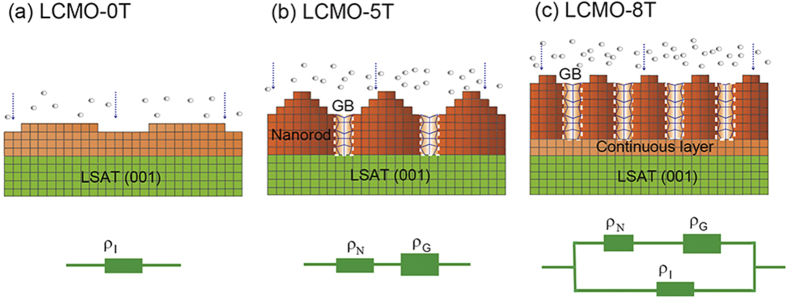
Schematic evolution of nanostructures in the LCMO thin films deposited under different high magnetic fields and a corresponding circuit analysis model. (**a**) The LCMO-0T planar film structure, (**b**) the LCMO-5T nanorod structure, (**c**) the LCMO-8T two-layer film structure. The red-brown color part represents LCMO planar film or nanorod, the gradient color part in the dashed box represents grain boundary between the nanorods.

The schematic evolution of nanostructures in the LCMO films deposited under different magnetic fields is shown in Fig. 4. For the LCMO-0T film, the stress between the film and substrate is not readily relaxed to form the defects such as misfit dislocations due to the small lattice mismatch between film and substrate^27^,^37^, which results in the epitaxial continuous growth with a layer-by-layer growth mode even for a film thickness of 220 nm (Fig. 4a). When a high magnetic field is applied in the film deposition processing, a number of defects will be produced at a critical thickness due to the confinement of surface diffusion and the increase of deposition rate due to the application of the high magnetic field. After the formation of the defects, the distribution of stress in the film is changed. The areas where the defects locate are stressed, whereas those areas between neighboring defects are unstressed or less stressed^27^. An island-like morphology will be formed with further film growth, on which the valleys correspond to the stressed areas whereas the peaks of the islands correspond to the unstressed areas. Most likely, these unstressed areas are energetically favorable for the epitaxial growth of the nanorods, and the stressed areas correspondingly form a high defect density at the grain boundaries between the nanorods^31^,^32^, as shown in Fig. 4b,c. For the LCMO-5T film, a nanorod structure with a highly disordered grain boundary between neighboring nanorods is formed, and a continuous layer cannot be almost observed (Fig. 4b). With further increasing magnetic field, for the LCMO-8T film, a continuous layer can be obviously observed due to the higher nucleation site density, and the dimension size of the nanorods is thinner with an almost unchanged width of grain boundaries between the nanorods compared with that of the LCMO-5T film (Fig. 4c). The nanorod structures and their geometrical arrangement in these films will affect the magnetic and LFMR properties of the films, as discussed in followings.

**Figure 5:**
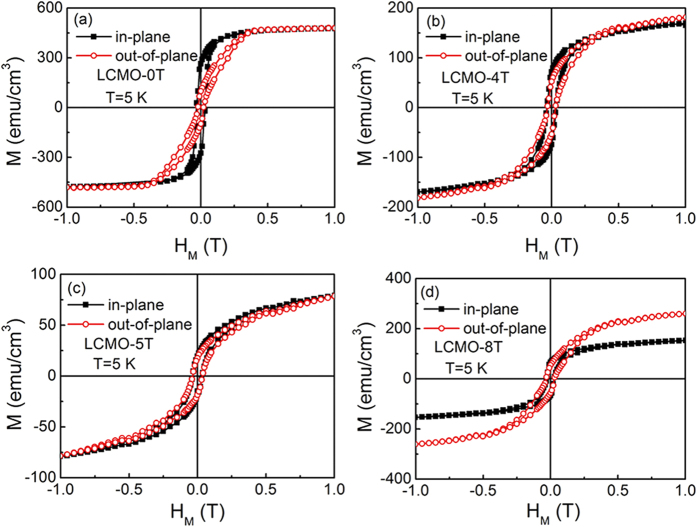
Hysteresis loops at 5 K of the LCMO films. The responses of the LCMO films were measured along the in-plane and the out-of-plane of the substrate: **(a)** LCMO-0T, **(b)** LCMO-4T, **(c)** LCMO-5T, and **(d)** LCMO-8T.

To understand the nature of the nanorod structures, the magnetic and transport measurements have been performed. The in-plane and out-of-plane hysteresis loops of the LCMO films at 5 K are shown in Fig. 5. The magnetic parameters extracted from the loops are listed in Table 1. It is noted that the variable tendencies of the in-plane and out-of-plane coercive fields show a rotation of easy axis of magnetization. The LCMO-0T film shows an in-plane easy magnetization (Fig. 5a), but the LCMO-8T film shows an out-of-plane easy magnetization (Fig. 5d). Vertical nanostructures have the great advantage over planar films in strain control because the strain is controlled by the large area vertical interfaces and not the single interface with the substrate^11^. It can be understood that the rotation of easy axis of magnetization is strongly related to the film structure. On the basis of the nanostructure model in Fig. 4, the LCMO-0T film has a planar film structure, showing an in-plane easy magnetization due to the weak lattice strain between the film and the substrate^14^,^38^. For the LCMO-5T and LCMO-8T films consisting of the nanorod structures, vertical strain plays a dominant role in property enhancements due to the vertical interface effect^11^, which results in the rotation of easy axis of magnetization. Furthermore, the vertical interface area increases with the decrease of the dimension size of the nanorods^9^,^12^,^13^, leading to a further out-of-plane easy magnetization. Thus, the LCMO-8T film shows a further enhanced out-of-plane easy magnetization compared with that of the LCMO-5T film. On the other hand, the highly disordered areas at the grain boundaries between the nanorods can also play an important role in the variation of magnetic anisotropy. The disordered areas with plenty of defects can give rise to structure disorder, chemical disorder and spins disorder^39^,^40^, leading to the increase of the pinned interface area, which will result in the increase of coercive field^16^. The disordered areas are also determined by the vertical interface area. As above-mentioned, the magnetic anisotropy of the derived LCMO films is strongly correlated to the vertical interface area of the nanorod structures, whereas the vertical interface area increases with the decrease of the dimension size of the nanorods. Namely, the magnetic anisostropy is highly dependent on the dimension size of the nanorods. From Table 1, the saturation magnetization of the derived LCMO films sharply drops firstly, and then reversely increases at 8 T. Initially, due to the increase of deposition rate and the enhancement of the confinement of surface diffusion, the defects such as misfit dislocations and two-dimensional islands are largely occurred, which will lead to a sharp drop of the saturation magnetization. For the LCMO-5T film, due to the formation of nanorods the vertical interfacial area is much larger than the substrate area. The additional interfacial energy will be released at the grain boundaries between the nanorods, and plenty of defects will be produced, which further result in a lower saturation magnetization. Meanwhile, although the released interfacial energy forms the disordered areas at the grain boundaries between the nanorods, the degree of structural perfection of the nanorods can be also simultaneously improved. The interfacial energy is released more thoroughly, the higher crystallinity of the nanorods. With further increasing magnetic field during film growth process, the dimension size of the nanorods reduces, and the vertical interface area increases, which favors the release of interfacial energy, and a higher crystallinity of the nanorods will be obtained, which are confirmed by HRTEM images and XRD results (not shown here). Thus, for the LCMO-8T film, the saturation magnetization increases reversely due to the improvement of the crystallinity of the nanorods. In addition, the additional continuous layer also contributes partly to the increase of the saturation magnetization.

**Figure 6:**
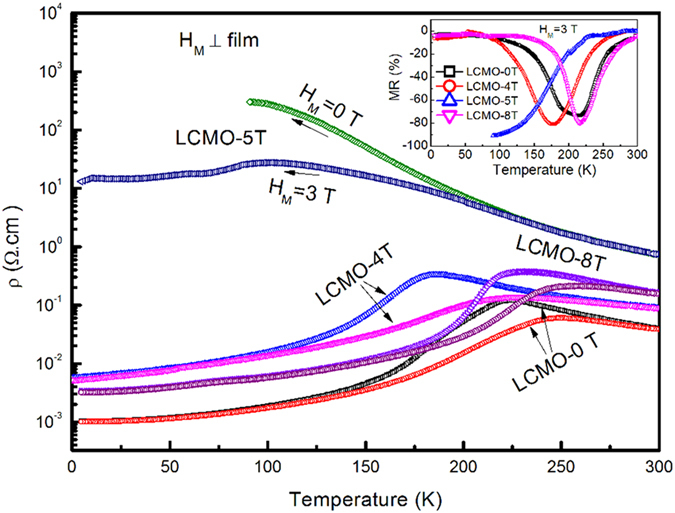
Temperature dependence of resistivity in the LCMO films measured at *H*_*M*_ = 0 and 3 T, respectively. The inset shows temperature dependence of MR in the LCMO films under *H*_*M*_ = 3 T.

**Figure 7:**
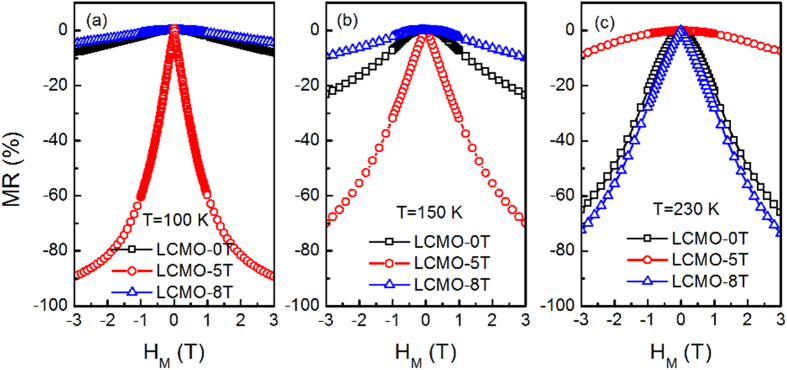
Magnetic field dependence of MR in the LCMO films at (**a**) 100, (**b**) 150 and (**c**) 230 K, respectively.

Figure 6 shows the temperature dependence of the resistivity for the derived LCMO films. It is interesting to observe that the resistivity of the LCMO-5T film largely increases and the insulator-to-metal transition is disappeared at *H*_*M*_ = 0 T, whereas the insulator-to-metal transition temperature *T*_*MI*_ is about 210 K for the LCMO-0T film. Moreover, an insulator-to-metal transition under 3 T at about 100 K is clearly observed for the LCMO-5T film, with a large negative magnetoresistance of –91% (inset of Fig. 6). Figure 7 shows the magnetic field dependence of MR. As shown in Fig. 7a,b, the MR of the LCMO-5T under 3 T, compared with that of the LCMO-0T, is sharply improved from −10% to −90% at 100 K and −20% to −70% at 150 K, respectively. Especially, a LFMR of -36% under 0.5 T at 100 K can be achieved for the LCMO-5T film. It is noted that these LFMR values are an enhancement as compared to the polycrystalline nanophasic LCMO (−33.5% vs. −16.3% at 1 T and 150 K)^41^, the LCMO: LaMnO_3_ composite (−26.6% vs. −25% at 0.3 T and 100 K)^42^ and the MgO/LCMO core-shell nanowires grown by PLD (−62% at 1 T and 100 K vs. −35% at 1 T and 170 K)^43^. Such a significant enhancement in LFMR is attributed to the enhanced carrier scattering at the grain boundaries between the nanorods^8^,^9^,^44^. The resistivity of the LCMO-8T film is slightly enhanced as compared with that of the LCMO-0T film, and the MR of the LCMO-8T film under 3 T is only slightly enhanced (-80% at 217 K) as compared with that of the LCMO-0T film (-74% at 217 K), which is also confirmed from the magnetic field dependence of MR at 230 K, as shown in Fig. 7c. The absence of obvious enhancement in MR and LFMR for the LCMO-8T film can be attributed to the appearance of the continuous layer adjacent to the substrate, which will lead to the electrical current passing through the continuous layer in spite of the nanorod layer in the resistivity measurement.

According to the experimental results above, it is confirmed that the vertical nanorod structures play an important role in regulating the magnetoresistance properties of the derived films. That is, tailoring the dimension size of these nanorod structures and their geometrical arrangement by the strength of magnetic field, as depicted in Fig. 4, can effectively change the conducting networks. Ning *et al.* have proposed the series parallel circuit model to understand the transport properties in LSMO: NiO nanocomposite films^16^. In our cases, based on the nanostructures of the derived films, the series parallel circuit model is also effective for the transport properties, as shown in Fig. 4. Ideally, the LCMO-0T planar film can be depicted as an intrinsic low-resistivity (ρ_I_).The LCMO-5T film can be regard as a series of low-resistivity (ρ_N_) nanorods and high-resistivity (ρ_G_) thin GB layers, then the effective resistivity can be described by ρ ≅ ρ_N_ + L^′^/L ρ_G_, where L is the dimension size of the nanorods, and L^′^ is the width of the grain boundaries between the nanorods^9^. The resistivity of this film is determined by the high-resistivity of grain boundaries between the nanorods. It is noted that the L^′^/L represents the LFMR effect can be tuned by tailoring the dimension size of the nanorods. Therefore, the LFMR will be further enhanced due to the changes of vertical nanostructure sizes. The LCMO-8T film can be taken as a parallel of a high-resistivity nanorod layer with a low-resistivity LCMO continuous layer, and the effective resistivity can be given by 1/ρ = A (*H*) / [ρ_N_ + L^′^/L ρ_G_] + B(*H*) /ρ_I_, where A(*H*) and B(*H*) are the weight factors depending on the magnetic field^16^. Since ρ_I_ « ρ_G_, the LFMR of the LCMO-8T film is almost in line with that of the LCMO-0T film. Based on the experimental and analysis results, it is confirmed that the LFMR properties can be tuned by tailoring of vertical nanorod structures with the application of an appropriate high magnetic field, which will provide a unique route to manipulate the LFMR, especially for the films on substrates with small lattice mismatch.

## Conclusion

Vertical aligned LCMO nanorod thin films have been obtained by high magnetic field assisted pulsed laser deposition method developed by ourselves, and the nanostructure nature of the LCMO films has been investigated. Under the applied magnetic fields in thin film processing, the nanostructures of the LCMO thin films can be easily tuned from epitaxially planar film growth to vertical nanorod structures with tunable dimension size with applied fields. The associated magnetic and transport properties are highly dependent on the nanorod structures. It is found that the magnetic anisotropy is strongly correlated to the dimension size of the nanorods. Furthermore, a significantly enhanced LFMR of −36% under 0.5 T at 100 K has been obtained for the LCMO-5T film due to the enhanced carrier scattering at the grain boundaries between the nanorods. A series parallel circuit model is proposed to understand the vertical nanostructure tailoring effects on the LFMR values. The growth mechanism of the nanorods has also been discussed, which can be attributed to the changes of plume dynamics, adatom surface diffusion, and nucleation as well as the growth mode induced by the applied high magnetic fields. The results will provide a unique route in fabricating and tailoring of vertical nanostructures in complex oxide thin films by the application of a high magnetic field in pulsed laser deposition processing.

## Methods

The LCMO films were deposited for 30 minutes under an oxygen pressure of 0.35 mbar at 650 °C. The laser energy and repetition frequency were fixed to 200 mJ and 5 Hz, respectively. After deposition, the films were *in situ* thermally treated for 20 minutes under the same temperature and oxygen pressure. In the process of both the deposition and thermal treatment of the LCMO thin films, high magnetic fields were applied perpendicularly to the film plane. The derived LCMO thin films were characterized by x-ray diffraction (XRD), field emission scanning electron microscopy and transmission electron microscopy. Magnetization measurements were performed by a Superconducting Quantum Interference Device Magnetometer (SQUID) made by Quantum Design and electrical transport measurements by a Physical Properties Measurement System (PPMS) by Quantum Design.

## Additional Information

**How to cite this article**: Zhang, K. *et al.* Vertical La_0.7_Ca_0.3_MnO_3_ nanorods tailored by high magnetic field assisted pulsed laser deposition. *Sci. Rep.* **6**, 19483; doi: 10.1038/srep19483 (2016).

## Figures and Tables

**Figure 1:**
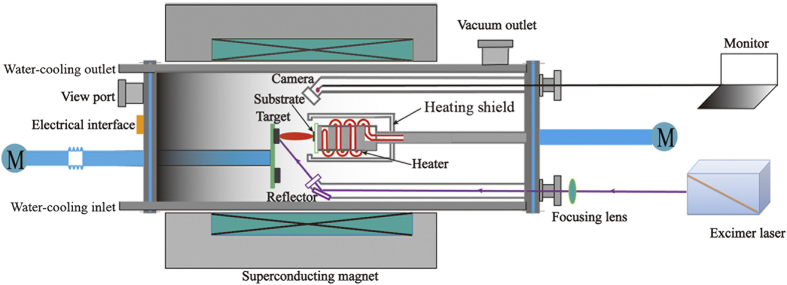
Schematic illustration of the high magnetic field assisted pulsed laser deposition system.

**Table 1:** Magnetic parameters of the LCMO films deposited at various magnetic fields.

Applied external magnetic field *H_D_* (T)	Coercive field *H_c_* (Oe)		Saturation magnetization *M_s_* (emu/cm_3_)
	In-plane	Out-of-plane	
0	321	214	470
4	354	318	183
5	360	360	78
8	276	332	262

*H*_*c*_ and *M*_*s*_ were performed at 5 K.
